# Characteristics of tobacco use among secondary school students: a cross-sectional study in a school in Valencia, Spain

**DOI:** 10.3389/fpubh.2023.1069294

**Published:** 2023-05-03

**Authors:** Joan Antoni Ribera-Osca, Francisco Carrion-Valero, Victor Martin-Gorgojo, Yolanda Rando-Matos, Carlos Martin-Cantera, Jose M. Martin-Moreno

**Affiliations:** ^1^Department of Health Valencia-La Fe, Health Care Center Alcàsser, Valencia, Spain; ^2^Pneumology Department, Hospital Clínico Universitario, Valencia, Spain; ^3^Medicine Department, Universitat de València, Valencia, Spain; ^4^Biomedical Research Institute INCLIVA, Hospital Clínico Universitario, Valencia, Spain; ^5^Orthopaedic Surgery and Traumatology Department, Hospital Clínico Universitario, Valencia, Spain; ^6^Primary Health Center Florida Nord, Direcció d'Atenció Primària Costa de Ponent, Catalan Health Institute, L’Hospitalet de Llobregat, Barcelona, Spain; ^7^Research Support Unit, Institut Universitari d’Investigació en Atenció Primària Jordi Gol (IDIAPJGol), Barcelona, Spain; ^8^Department of Preventive Medicine and Public Health, Universitat de València, Valencia, Spain

**Keywords:** smoking, tobacco, cigarette, adolescents, cross-sectional study, survey

## Abstract

**Introduction:**

Cigarette smoking is a significant public health problem, and it is essential to work actively with young people to limit the incorporation of this addiction. This study aimed to identify characteristics associated with tobacco use in adolescents in a real setting.

**Methods:**

Epidemiologic, cross-sectional study including secondary school students aged 12–17 years in the 1st, 2nd, and 3rd grades of “Joan Fuster High School” in the city of Sueca, Valencia (Spain). An anonymous, self-administered questionnaire was used to collect data on demographics, cigarette smoking history, alcohol consumption, nicotine dependence, and exposure to parental cigarette smoking.

**Results:**

The final sample of individuals surveyed included 306 students (50.6% females) with a median age of 13 years. The prevalence of cigarette smoking was 11.8% (13.5% in females and 9.9% in males). The mean age of cigarette smoking onset was 12.7 ± 1.6 years. Ninety-three students (30.4%) were repeaters, and 114 (37.3%) reported alcohol consumption. Significant factors associated with tobacco use were being a repeater (odds ratio [OR] 4.19, 95% confidence interval [CI] 1.75–10.55, *p* = 0.002), alcohol consumption (OR 4.06, 95% CI 1.75–10.15, *p* = 0.002) and parental cigarette smoking (OR 3.76, 95% CI 1.52–10.74, *p* = 0.007).

**Discussion:**

An operational profile of features associated with tobacco consumption was identified in the presence of parental cigarette smoking, alcohol consumption, and poor academic performance. Consideration of these factors could be useful in the operational design of cigarette smoking cessation interventions for young people in a context where there is a great need for better prevention and control of cigarette smoking.

## 1. Introduction

Smoking is considered one of the most relevant public health threats worldwide, and tobacco use is initiated primarily during adolescence (1, 2). The 2021 statistics of the US Centers for Disease Control and Prevention (CDC) on tobacco use among youth were striking: 4.0% of middle school students and 13.4% of high school students reported current use of a tobacco product (3). If cigarette smoking continues to increase at the current rate among this group of age, 5.6 million people bellow the age of 18 could die in the US of smoking-related illnesses (that is about 1 of every 13 Americans aged 17 years or younger who are alive today) (4). The 2019 report of the European School Survey Project on Alcohol and Other Drugs (ESPAD), based on 99,647 students (15- to 16-year-old) from 35 European countries, indicates that 9% of Spain’s students consume tobacco daily, with a prevalence of lifetime cigarette use of 41%, similar to the rest of 35 participating countries (5). These data are consistent with the 2021 report of the Spanish Observatory on Drugs and Addictions (6), in which 41.3% of secondary school students smoked tobacco once in their lifetime and 26.7% in the previous 30 days, with a mean age at smoking onset of 14.1 years. Surveys from other countries and regions have warned of the high prevalence of tobacco use among adolescent populations (7).

Cigarette smoking is considered to be initially triggered by personal experimentation and is often mistakenly conceived among adolescents as the transition to adulthood (8, 9). This could be explained by the Attitude Self-Efficacy (ASE) model (10), a behavioral change framework aimed at preventing risky behaviors, such as smoking. It comprises three determinants, including attitudes towards the behavior that are shaped by beliefs and the associated outcomes, subjective norms that are the expectations perceived from the immediate environment regarding the behavior, and self-efficacy, which relates to an individual’s expectations about their ability to engage in the health behavior.

In this respect, alcohol and tobacco are considered to be the most accessible drugs (5). In addition to the susceptibility to persistent addiction in adulthood, tobacco use during adolescence is associated with parallel alcohol consumption and cannabis use (8), with an increased hazard of depression and vascular damage (2). Moreover, social and environmental aspects can relate to smoking uptake. Thus, the probability of adolescents becoming smokers doubles if their peers smoke and triples if there is a favorable context towards smoking (11, 12). Kovacs et al. (13) identified low economic income, maternal alcohol consumption, parental smoking, and school dropout as determinants for smoking. Complementarily, higher tobacco consumption has been related to higher rates of school failure (14, 15). A link between tobacco use and parental smoking and/or perceived parental involvement (16), older age (17), alcohol intake, weak interest in school/poor academic performance, and being a smoker’s best friend (18) has also been described. Nevertheless, the level of dependence is usually low, which justifies early intervention in this age group (19, 20).

Considering the magnitude and seriousness of the problem, it is dissapointing the paucity of research and programs carried out in this field. Furthermore, results of interventions in adulthood once smoking has become firmly established are limited. With this in mind, the main aim of the study was to characterize the prevalence of tobacco use and the level of dependence among adolescents in a population of secondary school students aged 12 to 17 years, as well as to jointly assess the possible associations between tobacco use and sex, poor academic performance, alcohol consumption or parental smoking. The practical, underlying motivation of this study was the need to identify the features of adolescent smokers so that their characterization could help us to design more effective smoking prevention programs adapted to the defining traits of the target population of adolescent schoolchildren.

## 2. Materials and methods

### 2.1. Design and study sample

This was an observational, cross-sectional survey study of secondary school students aged 12 to 17 years enrolled at the “Joan Fuster High School” of the city of Sueca (with 26,617 inhabitants according to the 2021 census) located in the autonomous community of Valencia (Spain). The starting age for 1st ESO students was 12 years old, for 2nd ESO students it was 13 years old and for 3rd ESO students 14 years old. As the school course takes two calendar years, the ages have a range of one more year (i.e., 1st ESO 12–13 years old, 2nd ESO 14–15 years old and 3rd ESO 15–16 and up to a maximum of 17 years old). The socio-economic level of the areas covered by the institute corresponds to the working population, mainly in the industrial and agricultural spheres, and to a lesser extent in the economic area corresponding to services. This center was chosen for its accessibility and also for its size, since we could reach all enrolled students, and the total study sample was large enough to meet the needs set by the predetermined statistical power and the expected precision of the study.

Students who attended scheduled talks on tobacco use between October and November 2017, who agreed to participate, and completed the study questionnaire were eligible. These scheduled talks on tobacco were part of a parallel objective to the one that is the subject of this manuscript. In summary, the intervention consisted of a talk given by the medical professional who conducted the study to the participating students about the consequences of tobacco use, as well as specific training in social skills and social influence management in the face of tobacco use. This survey was framed as the initial baseline action of a quasi-experimental doctoral thesis research study of one of the authors (J.A. R-O.) (21), which aimed to assess the effect of a preventive intervention program against tobacco use compared to the current established interventions promoted by the local government administration.

The study was approved by the Ethics Committee of the Health Research Institute of Hospital Universitari i Politècnic La Fe of Valencia (code 2016/0599, approval date January 17, 2017). Written informed consent was obtained from parents/legal guardians, after which assent was obtained from the under-aged students.

### 2.2. Study procedures

An *ad hoc* questionnaire was designed. This was adapted from different sources, including a previous study from Zaragoza (Spain) on nicotine dependence among school students who were active smokers (22) and surveys from the annual activities (2010 to 2016) of the “smoke-free week” promoted by the Spanish Society of Family and Community Medicine (23, 24). The final questionnaire included a section with general aspects on age, sex, school year, being a repeater or not of any academic year, the student’s cigarette smoking history, attitudes towards cigarette smoking, and alcohol consumption, and a second section with questions related to passive cigarette smoking. The questionnaire was anonymous, self-administered, and had to be completed by participants in their classrooms without the presence of teachers. All students were invited to participate. The actual questionnaire (in Spanish) is available from the first author (J.A. R-O.) upon request.

Each student was classified according to tobacco use into daily cigarette smoker (smokes at least one cigarette every day), occasional cigarrete smoker (smokes at least one cigarette, but not every day, including the so-called “experimenters”), ex-cigarette smoker, and never a cigarette smoker. A current cigarette smoker was defined as the one who in the last month has smoked any number of cigarettes. On the other hand, an ex-smoker was defined as a person who been a cigarette smoker in the past, has not consumed tobacco cigarettes in the last 6–12 months. Alcohol use was also classified into current alcohol consumption (daily or almost daily), occasionally or only when going out to parties, and non-drinker. All categories of alcohol consumption were categorized into the group of alcohol consumption. In order to examine the level of passive cigarette smoking in the family environment, we inquired about the cigarette smoking habit of the parents, asking the students if their parents smoked around them, and they could answer: always or almost always, occasionally or never.

The level of nicotine dependence was evaluated according to the adaptations made by Clemente Jimenez et al. (22) to the Fagerström Test of Nicotine Dependence. Please refer to Supplementary material section for more details about it. In this adaptation, the language of the test is adapted to suit adolescents, and the items are recoded as so to ensure comparability with other tests. A score < 4 is considered low, 4–6 moderate, and 7–10 high dependence on nicotine.

### 2.3. Statistical analysis

Categorical data were expressed as frequencies and percentages, and continuous data as mean and standard deviation (SD) or median and interquartile range (IQR). We examined the subset of missing replies trying to verify any noticeable trend in the way data was missing and then removed the values only after verifying that they could be deleted without significantly distorting readings. Differences in studied variables between smokers and non-smokers were compared with the Chi-square test for categorical data and the Student’s t-test for continuous data. To assess factors associated with adolescent cigarette smoking, multivariate logistic regression model was fitted with covariates including sex, age, whether or not the patient was a repeater, alcohol consumption (categorized as yes/no), and parental cigarette smoking (categorized as yes/no). Odds ratio (OR) and 95% confidence intervals (CIs) were estimated. Statistical significance was set at *p* < 0.05. All statistical analyses were performed with the R statistical program (version 3.6.1) and the ordinal (2019.4–25) and clickR (0.4.32) packages.

## 3. Results

The total number of students from the 1st, 2nd, and 3rd grades at the “Joan Fuster High School” was 328. However, during the days in which the survey took place, 11 students were absent. Of the remaining 317 students who participated in the study, 11 provided incomplete questionnaires and were excluded from the analysis. Therefore, the final studied sample included 306 students, with a complete response rate to the questionnaire of 93.3%.

There were 151 boys and 155 girls, with a mean age of 13.4 ± 1.0 years. Almost 40% were in the 1st grade, and 30.4% were repeaters. Current smokers accounted for 11.8% of our sample, and 61.1% of smokers reported tobacco consumption on a weekly basis. The mean age at the onset of cigarette smoking was 12.7 ± 1.6 years. The level of nicotine dependence was low in 97.2% of the current cigarette smokers. Alcohol consumption was reported by 37.3% of participants, with 60.5% being occasional drinkers. More than 50% of students were exposed to passive cigarette smoking, with a rate of parental tobacco consumption of 54.1%. Salient characteristics of the study sample are shown in Table 1. Statistically significant differences between males and females in the distribution of study variables were not found.

**Table 1:** Demographic characteristics and data related to cigarette smoking, alcohol consumption, nicotine dependence, and parental cigarette smoking by sex.

Variables	Total students (*n* = 306)	Males (*n* = 151)	Females (*n* = 155)
Median age, years (IQR)	13 (13–14)	14 (13–14)	13 (13–14)
**Academic grade, *n* (%)**			
1st	113 (36.9)	60 (39.7)	53 (34.2)
2nd	112 (36.6)	48 (31.8)	64 (41.3)
3rd	81 (26.5)	43 (28.5)	38 (24.5)
Repeaters, *n* (%)	93 (30.4)	56 (37.1)	37 (23.9)
**Tobacco consumption, *n* (%)**			
Never smoker	214 (69.9)	109 (72.2)	105 (67.7)
Ex-smoker	38 (12.4)	27 (17.9)	29 (18.7)
Current smoker	36 (11.8)	15 (9.9)	21 (13.5)
Occasional (< 1 cigarette/week)	6 (16.7)	2 (13.3)	4 (19.0)
Weekly (≥ 1 cigarette/week)	22 (61.1)	11 (73.3)	11 (52.4)
1–10 cigarettes/day	7 (19.4)	3 (20)	4 (19.0)
11–20 cigarettes/day	1 (2.8)	0	1 (4.8)
> 20 cigarettes/day	1 (2.8)	0	1 (4.8)
Median age at cigarette smoking onset, years (IQR)	13 (12–14)	13 (12–13)	13 (12–14)
**Nicotine dependence, *n* (%)**			
Low	35 (97.2)	15 (100)	20 (95.2)
Moderate	0	0	0
High	1 (2.8)	0	1 (4.8)
**Alcohol consumption, *n* (%)**			
No	192 (62.7)	99 (65.6)	93 (60.0)
Yes	114 (37.2)	52 (34.4)	62 (40.0)
Daily/almost daily	2 (1.8)	1 (1.9)	1 (1.6)
Occasionally	69 (60.5)	29 (55.8)	40 (64.5)
Only when going out to parties	43 (37.7)	22 (42.3)	21 (33.9)
**Parental tobacco use (*n* = 303)**			
No	139 (45.9)	60 (40.3)	75 (48.7)
Yes (in the presence of adolescents)	164 (54.1)	89 (59.7)	79 (51.3)
In the presence of adolescents	72 (43.9)	30 (33.7)	42 (53.2)
Always/almost always	36 (22.0)	25 (28.1)	11 (13.9)
Occasionally	56 (34.1)	34 (38.2)	22 (27.9)

IQR: interquartile range (25th–75th percentile).

In the bivariate analysis, there were statistically significant differences between current smokers and non-smokers in terms of mean age (smokers were younger, 13.3 vs. 13.6 years respectively), percentages of repeaters (66.7% vs. 44.5%), alcohol consumption (75.0% vs. 56.1%), and parental tobacco use (83.4% vs. 53.1%), all of which were higher among cigarette smokers (Table 2).

**Table 2:** Bivariate analysis on the baseline characteristics of the secondary school students studied comparing the group of cigarette smokers with non-smokers.

Variables	Total students (*n* = 306)	Smokers (*n* = 36)	Non-smokers (*n* = 155)	*p*-value
Mean age, years ± SD	13.4 ± 1.0	13.3 ± 0.9	13.6 ± 1.1	<0.001
**Gender, *n* (%)**				
Male	151 (49.3)	15 (41.6)	136 (87.7)	0.422
Female	151 (50.6)	21 (45.6)	133 (85.8)	
Repeaters, *n* (%)	93 (30.4)	24 (66.7)	69 (44.5)	<0.001
Alcohol consumption, *n* (%)	114 (37.3)	27 (75.0)	87 (56.1)	<0.001
Parental tobacco use, *n* (%)	164 (54.1)	30 (83.3)	134 (53.1)	<0.001

In the logistic regression model, significant factors associated to tobacco consumption were being a repeater, alcohol consumption, and parental cigarette smoking (Table 3). Being a repeater (OR 4.19, 95% CI 1.75–10.55, *p* = 0.002), alcohol consumption (OR 4.06, 95% CI 1.75–10.15, p = 0.002) and parental cigarette smoking (OR 3.76, 95% CI 1.52–10.74, *p* = 0.007) were associated with an increased risk of tobacco consumption.

**Table 3:** Logistic regression analysis of the factors associated to tobacco consumption in the studied group of secondary school students.

Variables	Odds ratio (95% confidence interval)	*p*-value
**Gender**		
Females (reference)	1	
Males	0.55 (0.24–1.23)	0.149
Age, years	1.10 (0.70–1.72)	0.675
**Repeater**		
No (reference)	1	
Yes	4.19 (1.75–10.55)	0.002
**Alcohol consumption**		
Never (reference)	1	
Yes (daily/almost daily)	4.06 (1.75–10.15)	0.002
**Parental cigarette smoking in the presence of adolescents**		
No (reference)	1	
Yes	3.76 (1.52–10.74)	0.007

## 4. Discussion

Adolescents are particularly vulnerable to nicotine addiction and the adverse effects associated with tobacco smoking (25). Among adolescent cigarette smoking’ adverse effects is lung cancer, which has been firmly established. However, evidence is less cleared for other cancers, such as colorectal and breast cancer (26). Nonetheless, the prevalence of cigarette smoking of 11.8% among secondary school students in our study is high and consistent with data provided by the ESPAD project (5) and other studies (7, 27, 28). For this reason, it is essential to aim for a reduction in their initiation. The mean age of tobacco consumption onset in current cigarette smokers was 12.7 years, 19 months younger than that described in the ESPAD survey (14.1 years) (5). This may be due to a higher percentage of students in the first two grades of secondary school, which could have influenced the mean age of cigarette smoking onset. In fact, we are aware that we do not have age-specific surveys identical to those in our study, although those previously cited do include at least part of the consumption age range of our sample, so in this discussion we compare our estimates against those available from the reference surveys. However, there is no clear answer to explain these differences.

In the present study, there were a higher proportion of females who smoked (13.6%) compared with males (9.9%). However, this difference was not statistically significant. By contrast, other studies on adolescents have found that women smoke less than men (29, 30). Nevertheless, in a sample of 6,020 15- to 16-year-old pupils from 41 schools in England who completed an anonymous self-report survey, more females reported smoking, but males were more likely to be heavy smokers (31). Differences in smoking behaviors between male and female adolescent populations have been associated with numerous factors, including socioeconomic level and culture, the pressure of tobacco marketing, cigarette advertising and promotion, male masculinity, and feminine roles, perception of harm, expectations and self-control, body weight concerns, environmental pressure, and vulnerability to smoking after trying a single cigarette (30, 32–36). In addition, several studies indicate that women’s equality, misunderstood as the assumption of some traditional male roles, is often associated with poorer lifestyle habits, including tobacco con-sumption (25, 37).

Parental cigarette smoking is a strong and significant determinant for cigarette smoking by young people. In a meta-analysis of 58 studies, the relative odds of tobacco consumption in youth increased significantly if at least one parent smoked, especially if it was the mother or if both parents smoked (38). In a longitudinal analysis of data from 3,171 12- to 14-year-old students in 7 European countries allocated to the control arm of the European Drug Addiction Prevention trial, permissive parental norms toward cigarette smoking and alcohol use predicted adolescents’ use of illicit drugs, especially among boys (39). In the present study, the majority of students whose parents smoked (83.3% vs. 16.7% of non-smoker parents) consumed tobacco. Thus, it seems essential to recall the importance of the parental role model, which can greatly influence their children’s risky health behaviors. Moreover, alcohol consumption, parental tobacco consumption, and being a repeater of academic years were significantly associated with an increased likelihood of cigarette smoking in the regression model. Other studies have also shown that poor academic performance is associated with a greater probability of cigarette smoking initiation, more frequent cigarette smoking, a higher number of cigarettes smoked, and fewer attempts to quit smoking (40–42).

Given that adolescents who smoked were in the early stages of dependence, most of our participants under study (97.2%) had a low level of nicotine dependence. In the study by Clemente (22), whose population consisted of students in the age range of 10- to 17-years-old, low-to-moderate dependence rates were reported in a similar percentage of participants (86.6%). However, it has been shown that the first symptom of nicotine dependence can appear in some youths within days to weeks of the initiation of occasional tobacco use, often before the onset of daily smoking (43). The fact that symptoms of nicotine dependence may develop soon after initiation and/or at low levels of smoking suggests that novice adolescent smokers should not be neglected in smoking cessation interventions for early emerging symptoms (44).

Limitations of the study include the potentially limited external validity (generalizability to other schools in this district or other regions) due to the convenient sample, tobacco use defined by cigarettes only, and limited causality due to cross-sectional design. Other interesting variables, such as the use of electronic cigarettes or the assessment of biomarkers of tobacco exposure were not investigated. Moreover, self-report questionnaires may not always be reliable; although students were told in advance that the questionnaire was anonymous, it had to be completed without the presence of teachers in the classroom and truthfully because of the use of data for research purposes exclusively. This is probably the reason why participation among students was almost complete, being this fact noteworthy. However, we believe that the present results shed some practical facts to the current knowledge of adolescent cigarette smoking behavior and may help decision-making by authorities to develop preventive interventions for this population segment.

In conclusion, the present findings add evidence of the utmost importance of tobacco use in adolescence as a very relevant public health problem, especially because of an early age of onset. An operational profile of features associated with tobacco consumption was identified in the presence of parental cigarette smoking, alcohol consumption, and poor academic performance. Knowledge of this profile and the operational factors identified may be useful in designing cigarette smoking cessation in youth.

## Data availability statement

The datasets presented in this study can be found in online repositories, and can also be provided by the first author upon request. The names of the repository/repositories and accession number(s) can be found at: https://roderic.uv.es/handle/10550/76772.

## Ethics statement

This study was reviewed and approved by Ethics Committee of the Health Research Institute of Hospital Universitari i Politècnic La Fe of Valencia. Written informed consent to participate in this study was provided by the participants’ legal guardian/next of kin.

## Author contributions

JR-O, FC-V, and JM-M: conceptualization and methodology. JR-O and VM-G: formal analysis. JR-O, FC-V, VM-G, YR-M, and CM-C: investigation. JR-O: resources. FC-V and JM-M: writing. JM-M: supervision. All authors contributed to the article and approved the submitted version.

## Conflict of interest

The authors declare that the research was conducted in the absence of any commercial or financial relationships that could be construed as a potential conflict of interest.

## Publisher’s note

All claims expressed in this article are solely those of the authors and do not necessarily represent those of their affiliated organizations, or those of the publisher, the editors and the reviewers. Any product that may be evaluated in this article, or claim that may be made by its manufacturer, is not guaranteed or endorsed by the publisher.
