# Supplementary material for: Characteristics of tobacco use among secondary school students: a cross-sectional study in a school in Valencia, Spain
Source: Front Public Health. 2023 May 3;11:1069294. doi: 10.3389/fpubh.2023.1069294 (PMC10189142; doi:10.3389/fpubh.2023.1069294)
Supplement: Supplementary file 1 [file Table_1.docx]

**12 Supplementary material**

**Table 1 – supplementary material.** Translation of the adapted Fagerström Test of Nicotine Dependence for use in teenagers by Clemente Jimenez et al. (22)

| **Question** | **Answer** | **Points*** |
| --- | --- | --- |
| ***How long does it take from the time you wake up until you smoke your first cigarette?*** | Up to 5 minutes  6-30 minutes  31-60 minutes  More than 60 minutes | 3  2  1  0 |
| ***Do you smoke in forbidden places?*** | Yes  No | 1  0 |
| ***Which cigarette would cost you the most to suppress?*** | The first one  The one after meals  Any other | 1  0  0 |
| ***How much do you smoke?*** | Sporadic  Weekly  1-10/ 24 hours  11-20/ 24 hours  21-30/ 24 hours  31 or more/ 24 hours | 0  0  0  1  2  3 |
| ***When do you smoke more, in the morning or in the evening?*** | In the morning  In the evening | 1  0 |
| ***Do you smoke even if you are sick?*** | Yes  No | 1  0 |

** Score < 4 is considered low, 4-6 moderate, and 7-10 high dependence on nicotine.*
